# Evolutionary Dynamics and Epidemiology of Endemic and Emerging Coronaviruses in Humans, Domestic Animals, and Wildlife

**DOI:** 10.3390/v13101908

**Published:** 2021-09-23

**Authors:** Ariful Islam, Jinnat Ferdous, Shariful Islam, Md. Abu Sayeed, Shusmita Dutta Choudhury, Otun Saha, Mohammad Mahmudul Hassan, Tahmina Shirin

**Affiliations:** 1 EcoHealth Alliance, New York, NY 10001-2320, USA; ferdousjinnat90@gmail.com (J.F.); sharifislam@ecohealthalliance.org (S.I.); sayeed.dvm@gmail.com (M.A.S.); docshusmitadutta@gmail.com (S.D.C.); 2 Centre for Integrative Ecology, School of Life and Environmental Science, Deakin University, Burwood, VIC 3216, Australia; 3 Institute of Epidemiology, Disease Control and Research (IEDCR), Dhaka 1212, Bangladesh; tahmina.shirin14@gmail.com; 4 School of Veterinary Science, The University of Queensland, Gatton, QLD 4343, Australia; 5 Department of Microbiology, University of Dhaka, Dhaka 1000, Bangladesh; otun.saha@gmail.com; 6 Faculty of Veterinary Medicine, Chattogram Veterinary and Animal Sciences University, Chattogram 4225, Bangladesh; miladhasan@yahoo.com

**Keywords:** MERS-CoV, SARS-CoV, SARS-CoV-2, *Rhinolopoid*, zoonotic, surveillance, bats

## Abstract

Diverse coronavirus (CoV) strains can infect both humans and animals and produce various diseases. CoVs have caused three epidemics and pandemics in the last two decades, and caused a severe impact on public health and the global economy. Therefore, it is of utmost importance to understand the emergence and evolution of endemic and emerging CoV diversity in humans and animals. For diverse bird species, the Infectious Bronchitis Virus is a significant one, whereas feline enteric and canine coronavirus, recombined to produce feline infectious peritonitis virus, infects wild cats. Bovine and canine CoVs have ancestral relationships, while porcine CoVs, especially SADS-CoV, can cross species barriers. Bats are considered as the natural host of diverse strains of alpha and beta coronaviruses. Though MERS-CoV is significant for both camels and humans, humans are nonetheless affected more severely. MERS-CoV cases have been reported mainly in the Arabic peninsula since 2012. To date, seven CoV strains have infected humans, all descended from animals. The severe acute respiratory syndrome coronaviruses (SARS-CoV and SARS-CoV-2) are presumed to be originated in *Rhinolopoid* bats that severely infect humans with spillover to multiple domestic and wild animals. Emerging alpha and delta variants of SARS-CoV-2 were detected in pets and wild animals. Still, the intermediate hosts and all susceptible animal species remain unknown. SARS-CoV-2 might not be the last CoV to cross the species barrier. Hence, we recommend developing a universal CoV vaccine for humans so that any future outbreak can be prevented effectively. Furthermore, a One Health approach coronavirus surveillance should be implemented at human-animal interfaces to detect novel coronaviruses before emerging to humans and to prevent future epidemics and pandemics.

## 1. Introduction

The current novel coronavirus disease 2019 (COVID-19) was first identified as a cluster of pneumonia cases in China in December 2019 [1]. By March 2020, the disease had spread throughout the world; hence the World Health Organization (WHO) declared this virus as pandemic on 11 March 2020 [2]. The existence of the coronavirus was highlighted in 1950 when scientists identified the taxonomy of the virus [3]. The coronavirus is under the Coronaviridae family, which comprises of the Orthocoronavirinae and Torovirinae subfamilies [4]. The four generic categories of the coronavirus are alpha-coronavirus (α-CoV), beta-coronavirus (β-CoV), gamma-coronavirus (γ-CoV), and delta coronavirus (δ-CoV), which are under Orthocoronavirinae [4]. Of them, α- and β-CoV infect mammals, γ-CoV infects avian species, and δ-CoV infect both avian and mammalian species [5]. To date, a range of viruses, including Severe Acute Respiratory Syndrome coronavirus (SARS-CoV; SARS-CoV-2), Middle East Respiratory Syndrome coronavirus (MERS-CoV), and common cold viruses (e.g., 229E, OC43, NL63, and HKU1) under the Coronaviridae family have been identified [5,6]. The SARS and SARS-CoV-2 have been grouped under the same genus named Sarbecovirus and belong to the family Coronaviridae [7].

There are at least three structural proteins, namely a membrane protein (M), an envelope protein (E), and a spike protein (S) associated with the viral envelope. M and E proteins assemble the virus, whereas S protein mediates the virus entry into the host cell. Moreover, envelope-associated hemagglutinin-esterase protein (HE) is found in some coronavirus strains. The S protein is also the primary determinant of viral host range, tissue tropism, and a significant inducer of host immune responses [8]. Beyond these, coronaviruses pose a complex and diverse pattern of receptor recognition, which is an outstanding feature [8,9]. All the complex parts of the coronaviruses make them capable of adapting to new environments through mutation and recombination with relative ease and thus, can cause serious epidemic outcomes and one or a few residue variations in receptor homologs from different animal species can form critical barriers for cross-species transmissions [10,11,12].

Genome-wide point mutations or deletions/insertions in the accessory or spike protein genes are responsible for the change in the biological function of the virus. These genes often involve in or occur following CoV host-shift or tissue tropism change events. Thus, coronaviruses manifest an increased propensity for interspecies transmission. SARS and MERS-CoVs are the most crucial example of changing pathogenicity due to shifting host and genomic changes [11]. Several CoVs have been identified in different species, and some of them have already proven their pandemic potentials. There are many more in nature capable of causing a devastating impact on humans and animals [13,14,15,16]. Primarily, coronaviruses are responsible for causing respiratory, gastrointestinal, and central nervous system diseases in humans and other animals, thus threatening both health and economic loss in humans [8].

Earlier studies reported the possible spread of SARS-CoV from bats to palm civets to humans, and MERS-CoV from bats to camels to humans [17,18,19]. Illegal trading of palm civet, horseshoe bat (genus *Rhinolopus*), Chinese ferret badger (*Melogale moschata*), domestic cats, (*Felis domesticus*), and ferrets (*Mustela putorius furo*) in Chinese markets were claimed to be related to the transmission of the virus [20]. Though bats are thought to be the ancestor of the SARS-CoV-2, the exact ways of the spread are still obscure [21,22].

Under experimental conditions, coronaviruses demonstrated the capability to infect several animal species where ferrets [23], cats [23,24], hamsters [25], and rhesus macaques [26,27] showed evidence of virus replication in their respiratory tract. Additionally, cats and dogs in contact with infected humans have been identified as SARS-CoV-2 positive [28,29]. Therefore, there is occasional spillover evidence among human to animal species. The exact precursor for coronavirus is not established yet, but there is possibility of wild animal origin. Hence, for risk management and prevention of future pandemics, there is a need to study the virus through the identification of their ecology. Therefore, to identify the ancestors and close decedent of the coronaviruses is of utmost importance to explore the natural history and genomic analysis. Therefore, we conducted this study to understand the epidemiology and evolutionary dynamics of coronavirus diversity in humans, animals, and wildlife at ecosystem interfaces.

## 2. Materials and Methods

We conducted a detailed literature search using several keywords (Table 1). We searched the literature in Scopus (https://www.scopus.com/home.uri, accessed on 15 July 2021), Pubmed (https://www.ncbi.nlm.nih.gov/pubmed/, accessed on 15 July 2021), Web of Science (http://login.webofknowledge.com/, accessed on 15 July 2021), and Google Scholar (https://scholar.google.com/, accessed on 15 July 2021) on natural infection of coronaviruses in human and domestic animals and wildlife. We also searched grey literature on human and animal coronaviruses diversity. Then, we selected the literature based on the reporting of the natural infection among different animal species. We have presented our findings in tabular form. We collected the number of human SARS, MERS, and SARS-CoV-2 cases and presented the spatial distribution on the world map using ArcGIS software. In addition, we graphically showed the timeline of the emergence of different human and animal CoVs in the world.

We prepared several phylogenetic trees to show the ancestral relationship among (i) all the known human and animal CoVs, (ii) all the MERS viruses from animal and humans, (iii) all the SADS viruses, and (iv) all the SARS viruses from animals and humans. Initially, for every individual phylogenetic tree, an aggregate of more than 1000 (Creature and Human) accessible CoV whole genome sequences were retrieved from both NCBI GenBank (https://www.ncbi.nlm.nih.gov, accessed on 16 July 2021) and the Global Initiative on Sharing Avian Influenza database GISAID (https://www.gisaid.org, accessed on 16 July 2021) alongside the suitable reference successions. Genome quality, discreteness of their area, declaring time, land region closes by human animal interface reports, unpredictable model variety dates, close for the most part between every game plan, and arbitrary example assortment dates, were considered in the selection procedure. Later roughly 150/single tree genome arrangements were chosen on premise of their grouping quality, while genome successions having >5% NNNs and additionally bellow completely announced arrangements length were preclude from the examination. In addition, the number and ratio of sequences by country and family were determined based on the availability of number of sequences and the reported intensity of such viruses in a reported location. Furthermore, if there should be an occurrence of outgroup determination, we considered irregular choice course of groupings from the comparative kinds of receiving family. 

Representative sequences of CoV from human, animal, and wildlife were aligned using the Virus Pathogen Resource (https://www.viprbrc.org/, accessed on 18 July 2021) database and followed by phylogenetic analysis using MEGA 7.0 software as described by [30]. The neighbor-joining method [31] and the Kimura–Nei method [32] were considered for all the reported evolutionary relationship analyses where the bootstrap test (1000 replicates) is shown next to the branches. 

## 3. Results

**Figure 1:**
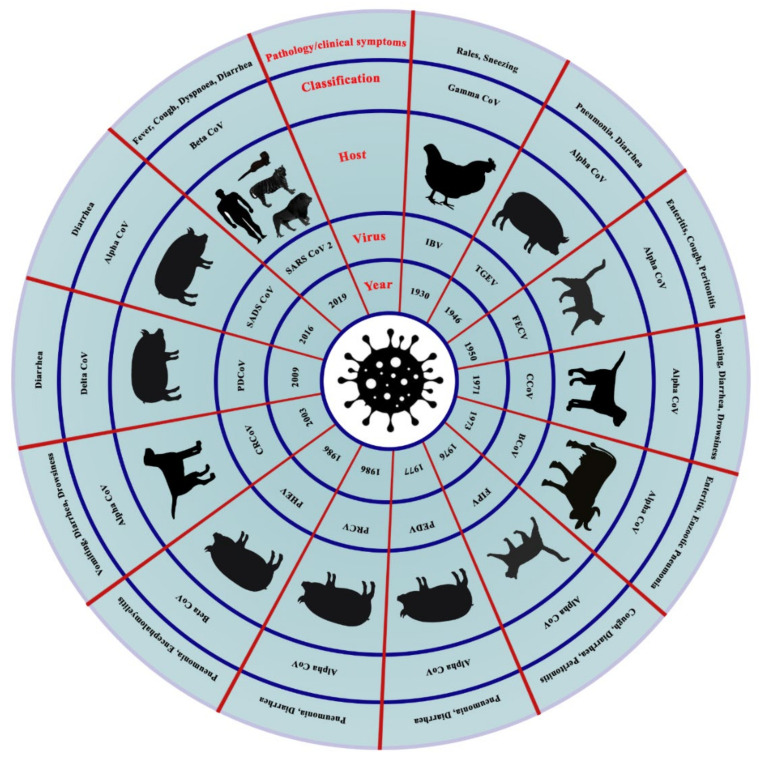
The emergence of animal coronaviruses in the world. The circles (inner to outer) indicate- the year of emergence, virus’s name, host species, the genus of coronavirus, and primary clinical signs. IBV: Infectious Bronchitis Virus; TGEV: porcine transmissible gastroenteritis coronavirus; FECV: Feline Enteric Coronavirus; CCoV: Canine Coronavirus; BCoV: Bovine Coronavirus; FIPV: Feline Infectious Peritonitis Virus; PEDV: Porcine Epidemic Diarrhea Virus; PRCV: Porcine Respiratory Coronavirus; PHEV: Porcine Hemagglutinating Encephalomyelitis Virus; CRCoV: Canine Respiratory Coronavirus; PDCoV: Porcine Delta Coronavirus; SADS: Swine Acute Diarrhea Syndrome Virus; and SARS: Severe Acute Respiratory Syndrome Coronavirus.

CoVs are single-stranded enveloped RNA viruses that can infect humans, other mammals, and avian species. CoV infections are often asymptomatic, but it mainly causes respiratory and digestive diseases in humans and animals, and occasionally affects the reproductive and nervous systems [33]. Based on evolutionary genetic analyses, a wide range of natural hosts has been reported for the virus (Figure 1).

### 3.1. Evolutionary Dynamics and Epidemiology of Coronaviruses in Livestock and Companion Animals

Several different CoVs have been identified in domestic animals such as cattle, horses, poultry, pig, etc., and companion animals such as dogs and cats (Figure 1). However, the virus had a long history from the year 1950 to 2019. In the year 1950, initially, the virus was identified among rodents where cattle were the intermediate hosts. Later, the viruses were identified among bats and mice as natural hosts. The large size of the RNA genome of CoVs expedites the emergence of new CoVs with altered antigenicity, virulence, tissue tropism, and/or host range [34,35]. Table 2 summarizes the coronavirus diversity in domestic animals and birds.

#### 3.1.1. Bovine Coronaviruses (BCoVs) in Ruminants

BCoVs, under the genus β-CoV, cause gastroenteritis and respiratory illness in calves aged under 3 weeks [36], winter dysentery in lactating cows [37], and shipping fever in fattening cattle. A BCoV-like enteric virus was isolated from a baby in 1994, and from then, it was a concern for public health [38]. Based on clinical signs, BCoV is divided into enteric BCoV (EBCoV), which causes diarrhea, and respiratory BCoV (RBCoV) which causes respiratory problems. EBCoV is further divided into EBCoV-CD, causing diarrhea in calves, and EBCoV-WD, causing winter dysentery in adult cattle [36].

**Figure 2:**
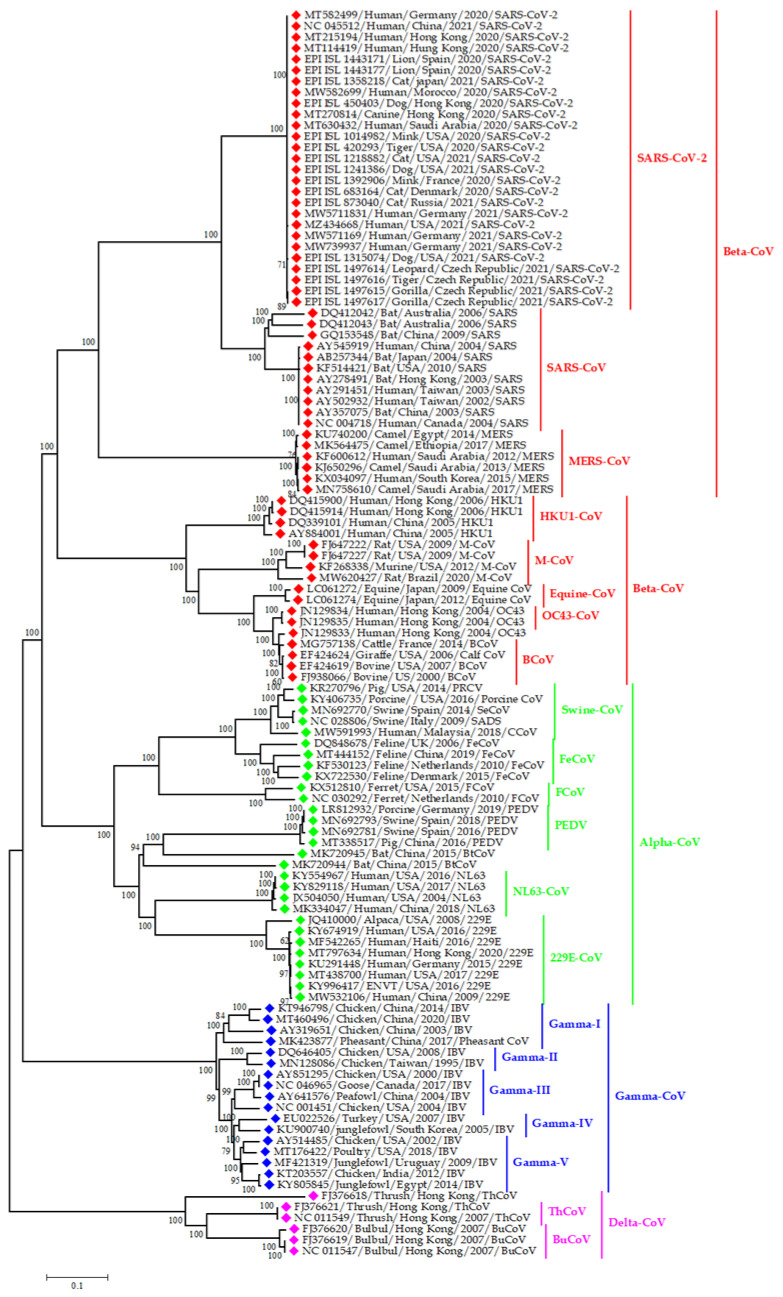
Phylogenetic analysis of CoV sequences from both animals and humans; green, red, blue, and pink color denotes alpha, beta, gamma, and delta coronaviruses, respectively. We constructed the phylogenetic tree representing the evolutionary relationship between all types of reported CoV in domestic animals, wildlife and human. This coronavirus family tree covering all four CoV genera; Alpha, beta, gamma and delta CoV. Both alpha and beta CoV genera segregated in various coronavirus strain like SARS-CoV, SARS-CoV-2, NL63, MERS-CoV, HKU1-CoV, OC43 etc. From this point of view, we have taken the various representative animals and human originated CoV sequences based on time and space for understanding the evolutionary relationship among all of the genera.

BCoVs are generally transmitted via the fecal-oral route or respiratory passage. This virus has a broad host range. Its host is not limited to cattle; it can also infect dogs, poultry, and giraffes [39]. In the case of buffalo, the first bovine-like CoV were detected using virus neutralization (VN) and hemagglutination inhibition (HI) assays in serum samples of water buffalo in Bulgaria [40]. Then, the virus was detected through RT-PCR from intestinal contents of buffalo with severe diarrhea between October 2006 and April 2007 in southern Italy [41]. The complete genome sequence of bubaline CoVs (BufCoV HKU26 strains B1-24F and B1-28F) was available in 2014 from Bangladesh, and it was 98–99% similar to BCoVs (Figure 2). To control BCoV, pregnant cattle are being vaccinated to provide maternal immunity to the newborn calf [42]. BCoV has 49.2–49.3% nucleotide closeness with SARS-CoV-2. Therefore, there is no chance of spillover of SARS-CoV-2 from bovines to humans (Figure 2).

#### 3.1.2. Swine Coronaviruses

**Figure 3:**
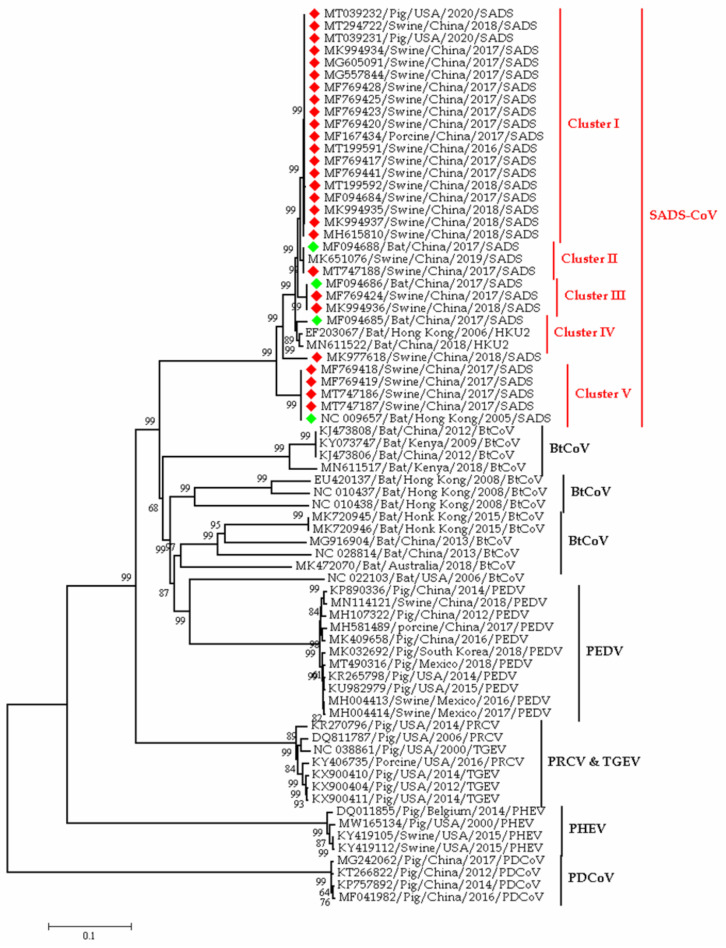
Phylogeny of swine coronaviruses detected to date in the world; the red blocks denote sequences from pigs, whereas green blocks denote sequences from bats. This phylogenetic tree representing the evolutionary relationship between representatives Alpha CoV originated from Swine host. The main purpose was to explain the evolutionally origin of SADS-CoV from Pig and Rhinolopoid bats. From this point of view, we have taken various representative- Bat and Pig originated SADS-CoV sequences on the basis of time and space. Besides, we also know that pig is infecting multiple types of alpha and beta coronaviruses like PEDV, PRCV, TGEV, PHEV and PDCoV. So, we selected representative sequences of swine infecting different types of coronaviruses based on different time and space to understand the phylogenetic relatedness of diverse swine coronaviruses.

Among the α-CoVs, porcine transmissible gastroenteritis coronavirus (TGEV), porcine epidemic diarrhea virus (PEDV), porcine respiratory coronavirus (PRCV), and swine acute diarrhea syndrome virus (SADS-CoV) infect pigs. Porcine hemagglutinating encephalomyelitis virus (PHEV) is under β-CoV, and porcine δ-CoV (PDCoV) is under δ-CoV, both of which have been detected in pigs [43]. These six CoVs usually infect pigs, but PDCoV can also infect badgers, calves, and cats [13]. In addition, PEDV can infect human cells [16], and they have a genetic resemblance to bat CoV (NC 022103) from the USA (Figure 3). Thus, PDCoV and PEDV have public health importance, as they can infect species other than pigs.

TGEV, PEDV, SADS-CoV, and PDCoV infect the gastrointestinal tract of suckling pigs and cause dehydration, anorexia, vomiting, and diarrhea [44]. From the name of PRCV, we can understand that it infects the respiratory system and exert mild symptoms. TGEV mutated and transformed into PRCV (Figure 3), and this mutation changes its affinity from the GI tract to the respiratory tract [43]. It was hypothesized that PDCoV’s origin was in birds, crossing the species barrier from birds to pigs [45].

In early 2017, a novel coronavirus related to HKU2, namely SADS-CoV was detected from neonatal piglets in South China, and it has been originated from horseshoe bats (*Rhinolophus*) (Figure 3). The phylogenetic tree in Figure 3 showed the clustering of SADS-CoV sequences with that of the bat from China and Hong Kong. It implies the ancestral origin of SADS in bats. However, the transmission of coronaviruses from bats to pigs is not yet confirmed, as all the SADS-CoV positive farms rear pigs in captivity. The SADS-CoV is an α-CoV within the subfamily Orthocoronavirinae [34,46]. A retrospective study on diarrheal disease samples in China indicated that the SADS–CoV has occurred since August 2016 [47]. In 2018, a separate strain of SADS-CoV was reported from Fujian province having 99.5% nucleotide identity with the reference strain of swine enteric α-CoV (SeACoV-p10) (Figure 2) [48].

The clinical symptoms of the SADS-CoV are mainly severe and acute diarrhea and vomiting, which is like other enteric coronaviruses of pigs [49]. This virus is highly pathogenic and causes high mortality in piglets. Piglets less than five days of age die due to loss of weight. SADS-CoV caused the death of 24,693 and 2000 pigs in 2017 and 2019, respectively [48]. Generally, the piglets die after 2–6 days of the onset of infection, while infected sows only suffer from mild diarrhea; most sows recover within two days [46]. Animal to animal transmission of SADS-CoV occurs through the fecal–oral route [48]. To date, there are no reports of SADS detection from any other provinces of China and/or the rest of the world [48]. Most recently, a novel δ-CoV, porcine CoV HKU15, has been detected in pigs [5].

#### 3.1.3. Coronaviruses in Camel

There are three CoV species detected in dromedary camels: MERS-CoV (β-CoV, group C), β-CoV 1/β1-HKU23-CoVs (group A), and human CoV 229E/camelid α-CoV [50,51]. MERS-CoV was first detected in a man in Saudi Arabia in 2012; it spread to Jordan and other countries outside the Arabian Peninsula [52,53,54]. Scientists found serological evidence, as well as RNA of MERS-CoV in dromedary camels [50,55]. These data strongly support the fact that dromedary camel is the reservoir of MERS-CoV. MERS-CoV and camelid α-CoV affect mostly camels aged between 6 months to 1 year. These two viruses were detected as 12.1% and 19.8%, respectively, in nasal swabs of the camel. Camelid α-CoV and camel β1-HKU23-CoV could not be detected in rectal swabs, and these viruses are shed via the respiratory tract of camels. Moreover, camelid α-CoV causes asymptomatic infections in Saudi Arabian camels and has genetic relation with HCoV 229E and alpaca CoV [15,56].

Mild symptoms develop in camels due to coronavirus, either in natural or experimental infection, including fever, nasal and lachrymal discharge, coughing, sneezing, and loss of appetite [57]. In the empirical study, infection is mainly restricted in the epithelium of the respiratory tract, and calves shed comparatively higher MERS-CoV than adults [58]. 

**Figure 4:**
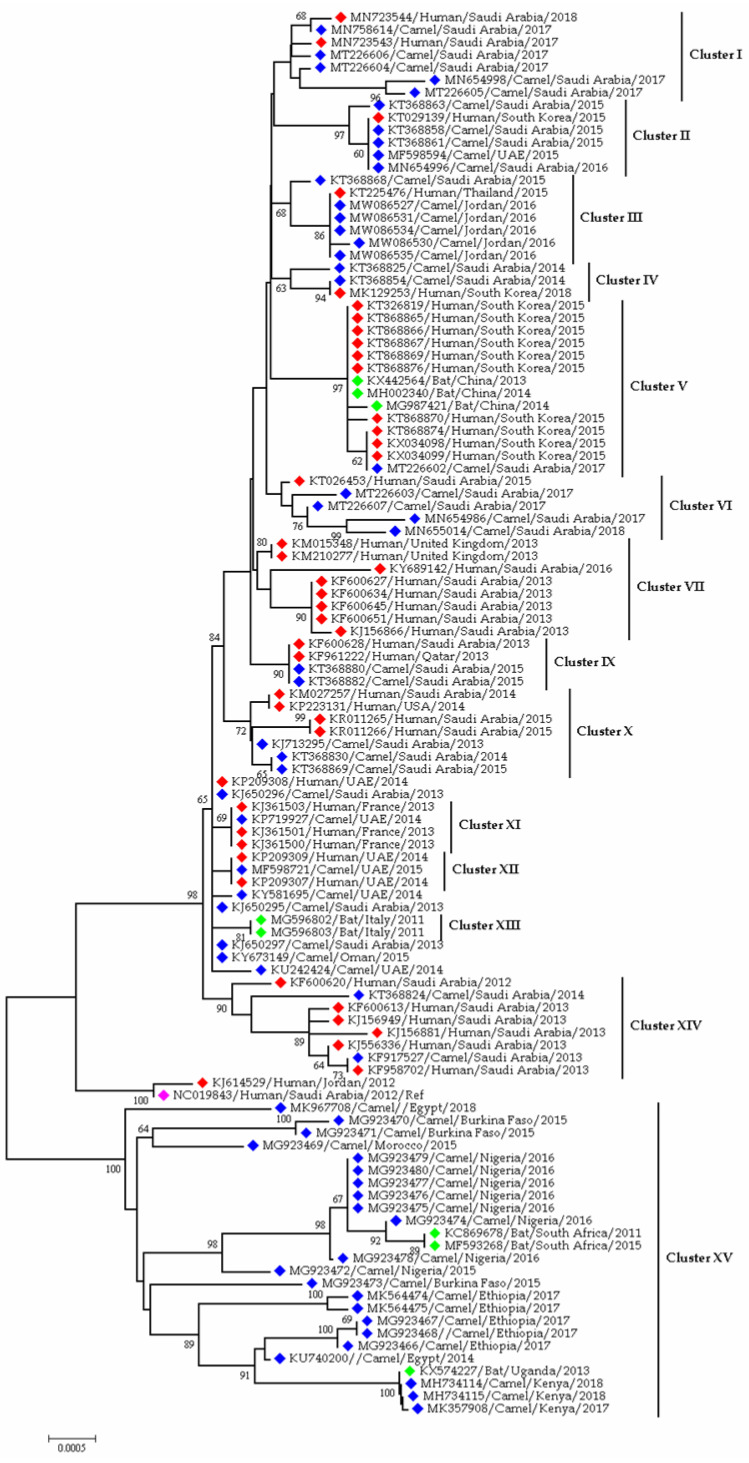
Phylogenetic analysis of MERS CoV sequences from human and camel; the red blocks denote human sequences, whereas blue blocks denote camel sequences; the green blocks denote bat virus sequences; and the magenta block refers to the reference sequence from humans of Saudi Arabia. This phylogenetic tree representing the evolutionary relationship of MERS-CoV sequences in human, camel and bat beta coronaviruses. Previous studies reported the evidence of camel to camel; camel to human and human to human spreading nature of the MERS-CoV. Therefore, we selected Gene bank deposited bat coronavirus sequences and MERS-CoV sequences from human and camels to understand the phylogenetic relatedness of MERS-CoV in human, camels and bats. From this point of view, we have picturized the bat- dromedary camel-human interfacial evolutionary relationship of MERS-CoV based on time and space.

Figure 4 depicts the phylogenetic relations among the MERS-CoVs from different countries. Camel and human strains from countries such as Saudi Arabia, Jordan, UAE, Qatar, and Oman are clustered due to having nucleotide similarities. Another interesting finding from the tree is the clustering of MERS-CoVs from camels of African countries such as Nigeria, Burkina Faso, Morocco, Ethiopia, Egypt, and Kenya. Moreover, MERS-like viruses were found in bats of Italy, China, Uganda, and South Africa. However, no MERS-CoV cases were detected in humans in African countries (Figure 4).

#### 3.1.4. Coronavirus Diversity in Birds 

To date, 108 avian species under 30 families and 15 orders have been found positive for coronaviruses [59]. Birds are ideal hosts for γ- and δ-CoV’s evolution and dissemination (Table 2) [5]. There are five ratified species in the genus γ-CoV, while the δ-CoV contains seven ratified species spread across three subgenera [59]. However, their co-existence in avian hosts does not favor recombination [5]. The γ-CoV, infectious bronchitis virus (IBV), is widespread in poultry and wild birds and was first detected in 1937 [60]. It is highly contagious and infects the respiratory and urogenital systems of poultry [61]. It causes sneezing, diarrhea, and reduces production [61,62]. IBV has also been detected in wildfowl (Anseriformes), waders (Charadriiformes), rock doves (Columbiformes), wild peafowl (Galliformes), Pied Heron (Pelecaniformes), and some passerine species (Passeriformes) (Figure 2) [63,64]. Farmers use live attenuated vaccines [62] for IBV prevention in poultry, but the constant emergence of novel serotypes makes it challenging to control.

Other species affected by avian γ-CoVs are turkey and pheasant. Moreover, CoVs were also isolated from peafowl, guinea fowl, partridge, and teal. All these viruses are similar to IBV. Thus, IBV’s host range may extend to species other than chicken. Recently, γ-CoVs have been identified in goose, mallard duck, pigeon, quail, peafowl, and partridge [65]. However, these viruses are not related to IBV [66]. Lately scientists have found three novel CoVs, bulbul CoV HKU11, thrush CoV HKU12, and munia CoV HKU13. Other novel δ-CoVs were found in different species of birds, i.e., white eye CoV HKU16, sparrow CoV HKU17, magpie robin CoV HKU18, night heron CoV HKU19, wigeon CoV HKU20, and common moorhen CoV HKU21 [5]. Quail δ-CoV was detected in UAE in 2018 for the first time and had similarities with swine and tree sparrows CoV. Quail CoV was also identified in Poland and had a genetic resemblance with the one from UAE [67].

CoVs in wild birds have been identified in several countries of the world [5,68,69,70,71]. The archived avian samples were tested in China for SARS CoV-2, but none of the samples was found positive [72,73]. It should be noted that SARS-CoV-2 has around 43.0–43.2% genetic similarity with IBV. Although only limited studies have been conducted on wild birds for coronaviruses, these viruses have been detected frequently from all continents of the world, including Antarctica [59].

#### 3.1.5. Coronavirus in Companion Animals (Dogs and Cats)

Canine coronaviruses consist of canine coronavirus (CCoV) and canine respiratory coronavirus (CRCoV). CCoV belongs to α-CoV, and CRCoV belongs to β-CoV. CCoV causes appetite loss, vomiting, diarrhea, and dehydration in dogs. Four strains of CCoV can exchange genes with TGEV. There may be possibility of interspecies transmission of viruses between dogs and pigs (Figure 2) [74]. The CCoV’s genotype II can bind with both canine aminopeptidase N (APN) and feline APN. This concept contradicts the general assumption that every virus utilizes a species-specific receptor [75]. Additionally, this also indicates possible cross-species transmission of CCoV. 

CRCoV causes cough and bronchopneumonia in canines. However, it may have a common ancestor with bovine CoV as they have nucleotide resemblance [76]. Few reports of recombinant canine CoVs are found. Therefore, it is of utmost importance to study the recombinant canine CoVs. SARS-CoV-2 and canine CoVs have 43.6–44% genetic similarity, meaning that canine CoVs are not the source of SARS-CoV-2 (Figure 2). Inactivated and attenuated canine CoV vaccines are available, but as the viruses cause subclinical infection in dogs, the use of the vaccine is not suggested [77]. 

Feline CoVs are αCoV; two biotypes of coronavirus are found in cats, feline enteric CoV (FECV) and feline infectious peritonitis virus (FIPV). FECV infects the cat’s intestinal cells and causes enteritis [78], whereas FIPV infects monocytes and causes systemic diseases. FIPV can cause lethal peritonitis and purulent granulomatous inflammation [79]. These two biotypes cannot be separated antigenically, morphologically, or serologically [80]. FECV mutated and changed its affinity from intestinal epithelium to monocyte [81]. There is a hypothesis that FECV and canine CoV are genetically recombined to form FIPV [82]. Therefore, it can be said that the change in host tropism or tissue tropism can be the result of mutation or recombination of feline and canine CoV. Similar to the canine CoV, feline CoV is unlikely to be the source of SARS-CoV-2, as they have only 43.3–43.6% nucleotide resemblance (Figure 2). At present, the vaccination against feline CoV is not suggested as there may be antibody-dependent enhancement (ADE) [77]. Advance studies should be conducted to develop a potent vaccine against feline CoVs.

The recently emerged SARS CoV-2 has been detected in 62 cats and 33 dogs in total [83]. In most cases, the pets had previous history of contact with COVID-19 positive humans [73,84]. Recently, the emerging alpha-variant (B.1.1.7) of SARS-CoV-2 was detected in dogs and cats in the USA [28].

#### 3.1.6. Other Coronaviruses

In October 2007, alpacas (*Vicugna pacos*) in California, USA, were showing acute respiratory disorder with fever and sudden death. Most alpacas suffering from the disease were pregnant. After that, similar cases were found on the east coast of the USA, but the authority could not identify the causative agent. Finally, a novel coronavirus was detected from the lung of an alpaca [56]. On the other hand, equine CoVs (ECoV) are relatively new compared to other CoVs in animals. It is a β-CoV, and only four complete genome sequences are available to date [85,86]. It affects adult horses [87] and causes fever, lethargy, anorexia, and, less frequently, colic and diarrhea [88]. ECoV’s prevalence is significantly lower (2–6% per year), but positive cases are higher in cold months (October to April). ECoV infection is more common in riding, racing, and show horses and less common in breeding animals. No licensed vaccine has been available against this virus until now.

### 3.2. Evolutionary Dynamics and Epidemiology of Coronaviruses in Wild Animals 

#### 3.2.1. Coronaviruses Diversity in Bats

Bats are natural hosts for α- and β-CoV evolution and dissemination [5]. More than 200 novel CoVs have been detected in bats over the world [89], and they are considered as host of SARS-CoV-related viruses [90]. SARS-CoV-related viruses were detected in Rhinolophus bats (Horseshoe bats) with almost 98% genetic similarity with SARS-CoV [91,92]. These bat-borne SARS-CoV-related viruses use the same ACE2 receptor on humans [93]. In addition to SARS-like viruses, bats are also host to evolutionary ancestors of MERS-CoV and MERS-CoV-related viruses [50]. Even the SADS-CoV, an important pathogen for pigs, was traced back to Rhinolophus bats due to the virus’ genome sequence identity of 98% to a bat CoV, HKU2 [46]. Similarly, bat CoVs have a distant relationship with HCoV 229E.

After the SARS-CoV-2 outbreak worldwide in 2020, scientists are relentlessly examining bat samples throughout the world. SARS-CoV-2-like viruses have been identified in different bat species such as *R. sinicus*, *R. stheno*, *R. pusillus*, *R. malayanus*, *R. affinis*, *R. shameli*, *R. cornutus*, and *R. acuminatus* from Thailand, Japan, Cambodia, and China [21,22]. Moreover, SARS-CoV-2 is thought to be originated from bats, as there are similarities between the spike (S) glycoprotein of SARS-CoV-2 with the S protein of bat CoV, RaTG13 [94]. Upon full genome sequence analysis, SARS-CoV-2 showed 96.2% genome identity with bat coronavirus, RaTG13 [95], directing that bat CoV and human SARS-CoV-2 might share the same ancestor.

#### 3.2.2. Coronaviruses Diversity in Pangolin

Captive Malayan pangolins (*Manis javanica*) in China were found to be infected with a β-CoV. The virus was novel, having resemblances with SARS-CoV-2. Pangolin-associated coronaviruses belonged to two sub-lineages of SARS-CoV-2 related coronaviruses with a resemblance in RBD of SARS-CoV-2 [96]. Moreover, the pangolin coronavirus genome shares 89% nucleotide and 98% amino acid resemblances with SARS-CoV-2 [97]. However, recently, a group of scientists tested 334 Sunda pangolins seized during illegal trading in Peninsular Malaysia and Sabah between August 2009 to March 2019. Tests were negative for coronavirus, which reflects that pangolins are not reservoir or intermediate hosts for SARS-CoV-2; instead, they might become infected by humans or any other animal species along with the trade network [98].

#### 3.2.3. Coronaviruses in Wild Felids

Feline CoV infects a variety of species, including cheetahs (*Acinonyx jubatus*). FIPV, the αCoV, can cause similar symptoms in cheetahs and domestic cats. When a cheetah is exposed to domestic cats or prey exposed to contaminated foods, the cheetah is exposed to the FIPV [99,100]. Cheetahs are very susceptible to FIPV [101]. FIPV can infect other felid species, such as pumas (*Puma concolor*) and African lions (*Panthera leo*) [102,103]. Pumas are also susceptible to feline enteric CoV (FeCV). Almost 28% of pumas had antibodies against feline CoV in California [102]. CoV outbreaks were recorded in wild cats, *F. silvestris*, from 1982 to 1992 in Scotland [104].

Enteric canine CoV causes sporadic infection in wolves (*Canis lupus lupus*) [105,106], whereas in red foxes (*Vulpes vulpes*), the CCoV causes enteritis [107]. Raccoon dogs (*Nyctereutes procyonoides*) and masked palm civets (*Paguma larvata*) can act as intermediate hosts of SARS-like CoV strains [108]. FCoV type II and CCoV have infected spotted hyenas (*Crocuta Crocuta*) but no signs were described [109,110]. Silver-backed jackals (*Canis mesomelas*) have detectable CoV in feces, but no lesions were defined [110].

#### 3.2.4. Coronaviruses in Miscellaneous Wild Animals

BCoV-like viruses can infect captive and free-range wild ruminants such as sambar deer (*Cervus unicolor*), white-tailed deer (*Odocoileus virginianus*), sika deer (*C. nippon yesoensis*), caribou (*Rangifer tarandus*), and water deer (*Hydropotes inermis*). These viruses can cross-react with BCoV [41]. These viruses cause gastroenteritis in neonatal calves and lactating cattle; respiratory disease in growing and steer calves. Waterbuck antelopes (*Kobus ellipsiprymnus*) show winter dysentery [111], whereas giraffes (*Giraffa camelopardalis*) and llamas (*Lama lama*) show gastroenteritis after exposure to the BCoV-like virus infection [39,112]. Similarly, elk or wapiti (*C. elephus canadensis*) can also harbor BCoV-like viruses that cause mortality in neonates under 3 weeks of age, and in calves between 3 weeks to 1 year of age [41]. Other species, such as musk oxen (*Ovibus moschatus*), sitatunga (*Tragelaphus spekei*), wisent (*Bison bonasus*), Himalayan tahr (*Hemitragus jemlahicus*), and Nyala (*Tragelaphus angasii*) showed symptoms such as watery or bloody diarrhea/dysentery after being infected with BCoV-like viruses [111,113]. 

Hedgehog coronavirus 1 is commonly known as Erinaceus CoV (EriCoV) but does not cause disease in European hedgehogs (*Erinaceus europaeus*). Hedgehogs may be the reservoir of the virus [114]. Similarly, amur hedgehogs (*E. amurensis*) are the reservoir of hedgehog CoV HKU31 (Ea-HedCoV HKU31) [115]. Recently, a novel αCoV, Wencheng shrew virus (WESV), was found in Asian house shrew (*Suncus murinus*), but it also did not produce any disease [115]. Bubaline CoVs (BuCoVs) are genetically related to BCoV and infect water buffalo calves. Though BCoV and BuCoVs are similar, their biological properties are somewhat different [116]. Captive wild animals such as tigers, lions, gorillas, pumas, cougars, leopards, and otters were infected by SARS-CoV-2 from asymptomatic human caretakers [117]. Asiatic lions in India were infected by the emerging delta variant (B.1.617.2) of SARS-CoV-2 [118].

#### 3.2.5. Coronaviruses in Ferret and Mink

Ferret enteric CoV (FRECV), under the genus α-CoV, can cause epizootic catarrhal enteritis (ECE) in ferret. In particular, the MSU-2 and No22 strains of FRECV infect ferrets clinically [119,120]. Virus-like substances can be observed in feces, and the virus can be found in the intestine of the ferrets. However, no RNA was detected in the GI tract or spleens of the ferret [121]. Another important α-CoV is ferret systemic CoV (FRSCV) which affects several systems and causes granulomatous lesions [120,121]. The virus affects the GI tract, the brain, and other internal organs [122,123].

Similar to ferret, mink can also be infected by ECG [124]. The disease was first reported in 1975, and then several countries such as the USA, Canada, Scandinavia, China, and Russia reported the virus in millions of minks. Minks show anorexia and mucus diarrhea within 2 to 6 days of infection. In 2011, a suspected CoV outbreak affected masses of minks [125]. Most recently, mink infection by SARS-CoV-2 has been reported from hundreds of farms from eight countries in Europe: Denmark, the Netherlands, Greece, Sweden, Spain, Lithuania, France, and Italy [126]. The virus causes respiratory disease with typical viral pneumonia in histopathology, which can be transmitted from mink to mink. The infection in minks was initiated by humans, and then a specific mink variant emerged. Then, mink transmit the virus to humans again [127]. Not only farmed mink but also wild minks were found to be infected by SARS-CoV-2 though showed no symptoms [128]. The possible source of infection to wild mink is considered to be wastewater in one case [128]. Even twelve feral cats and two dogs were infected by SARS-CoV-2 in the Netherland. The study concluded that the feral cats were infected from minks, but whether the source of infection for the dogs was mink or humans was not clear [129,130]. 

#### 3.2.6. Coronavirus in Rodents

Viruses that infect rodents are known as murine CoV (Figure 2). The murine CoV group is under the genus betacoronavirus. CoVs evolution, especially the subgenus embecoviruses under the genus betacoronavirus, can be significantly attributed to rodents. The embecoviruses mainly affect animal species [35] and humans. HKU1 is the human coronavirus under embecovirus. There are two murine CoVs, mouse hepatitis virus (MHV) and rat CoVs (RCoV). MHV was first identified in 1949, but it causes mainly asymptomatic infection [131]. There are several strains of the virus, and the severity of infection relies on age, immunity, and genotype of hosts [131]. Mainly two types of MHV strains are found; one replicates in nasal epithelium and another one with an affinity to GI tract epithelium [131].

The RCoV are of two types, sialodacryoadenitis virus (SDAV) and Parker’s RCoV (PRC). SDAV is distributed worldwide in laboratory rats [132]. SDAV is more infectious, but less lethal [131]. SDAV and PRC can cause cross-reactivity with MHV, BCoV, and HCoV-OC43 [133]. PRC affects the respiratory tract, whereas SDAV affects salivary and lacrimal glands [131,134]. Recently scientists discovered some new CoVs: Lucheng Rn rat coronavirus (LRNV), Longquan Aa mouse coronavirus (LAMV), Longquan Rl rat coronavirus (LRLV), and HKU24 in rodents in Europe and China. These viruses cause asymptomatic infection in rodents [135]. Another rat CoV, HKU24, has been detected in China which is novel. However, this virus does not cause any disease in rats [136]. Coronavirus-like symptoms can also be found in guinea pigs [137].

#### 3.2.7. Coronavirus in Non-Human Primates

The common cold-causing pathogen in humans, the β-CoV HCoV-OC43, can affect other non-human primates such as wild chimpanzees (*Pan troglodytes verus*). Chimpanzees have suffered from sneezing and coughing due to HCoV-OC43 infection in Cote d Ivoire [138]. Recently, gorillas have been infected by SARS-CoV-2 in a zoo by an asymptomatic human caretaker [117].

#### 3.2.8. Coronavirus in Marine Mammals

Only α- and γ-CoVs have been described in marine animals, but no β-CoV was recovered from them [139,140]. To date, the following CoVs have been detected in marine mammals: the Harbor Seal α-CoV (Bossart and Schwartz, 1990), Pacific Harbor Seal γ-CoV [141], Beluga Whale γ-CoV [142], and the Bottlenose Dolphin γ-CoV [143]. The α- and γ-CoV infection in seals cause respiratory secretions and are possibly associated with pneumonia [141,144]. In cetaceans, the viruses cause only respiratory disease [142]. However, there is no confirmed pathology of coronavirus infection [145], and unfortunately, no marine γ-CoV has been cultivated to date [145].

### 3.3. Epidemiology and Evolutionary Dynamics of Emerging Coronaviruses in Humans

**Figure 5:**
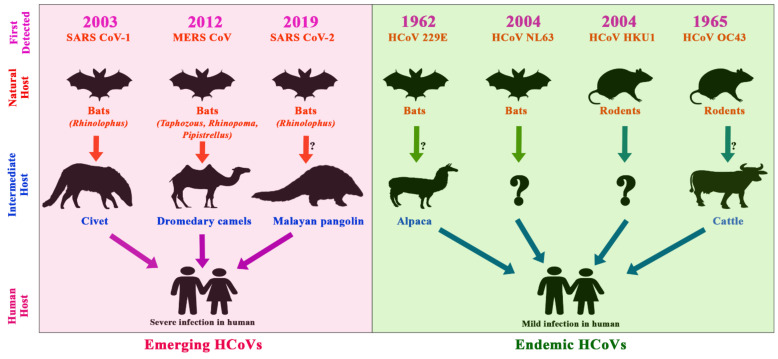
Timeline of the emergence of human CoVs, their reservoirs, and intermediate hosts; the pink shaded area depicts emerging coronaviruses, whereas the green shaded area depicts the endemic coronaviruses. The arrows show the transmission route of the viruses from animal to human through intermediate hosts.

To date, seven HCoVs have been identified, namely HCoV-229E, NL63, OC43, HKU1, SARS-CoV, MERS-CoV, and the latest, SARS-CoV-2 [146]. Almost all the reported HCoVs have intermediate hosts; events of reverse zoonosis were also identified (Figure 5).

The first human coronavirus was 229E, identified in 1966. After one year, another, OC43, was detected in a typical cold patient [17,147]. HCoV-NL63 and -HKU1 were discovered later in 2004 and 2005, respectively [148,149]. HCoV-229E and -NL63 belong to αCoV, whereas HCoV-OC43 and -HKU1 belong to β-CoV. HCoV-229E and -NL63 shared 65% genetic similarity. These HCoVs are endemic and contribute to one-third of the common cold or mild to moderate upper-respiratory tract infections in the human population globally [150]. These viruses are involved in 20% of patients’ respiratory acute illnesses [151]. However, they can cause severe disease in immunocompromised individuals and children [152]. Additionally, the viruses do not cause gastroenteritis yet, but they can be found in stool samples [153]. OC43 can sometimes be related to chronic demyelinating disease and acute encephalomyelitis [154].

**Figure 6:**
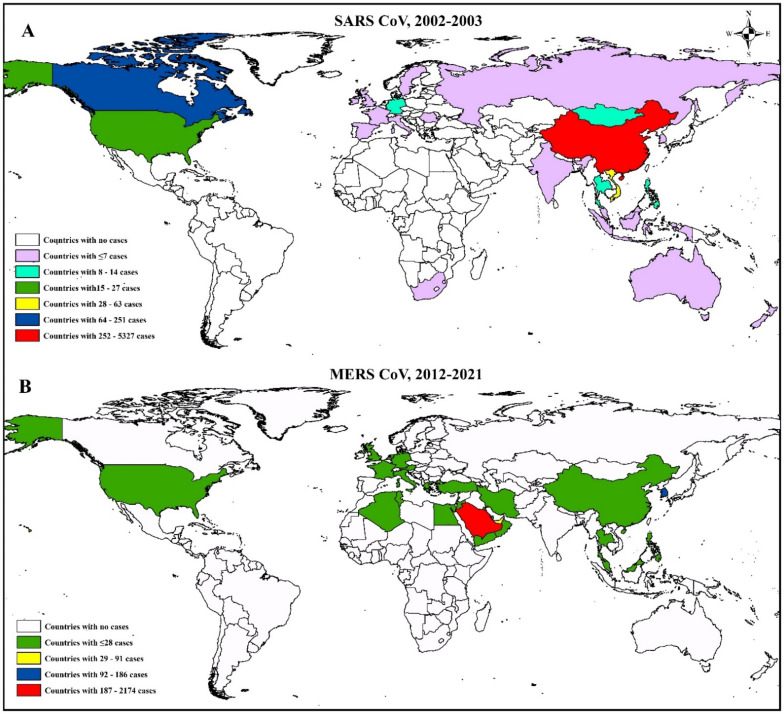
Geospatial distribution of SARS-CoV and MERS-CoV globally; A. SARS-CoV and B. MERS-CoV case distribution globally; the marked red areas have the highest number of cases.

**Figure 7:**
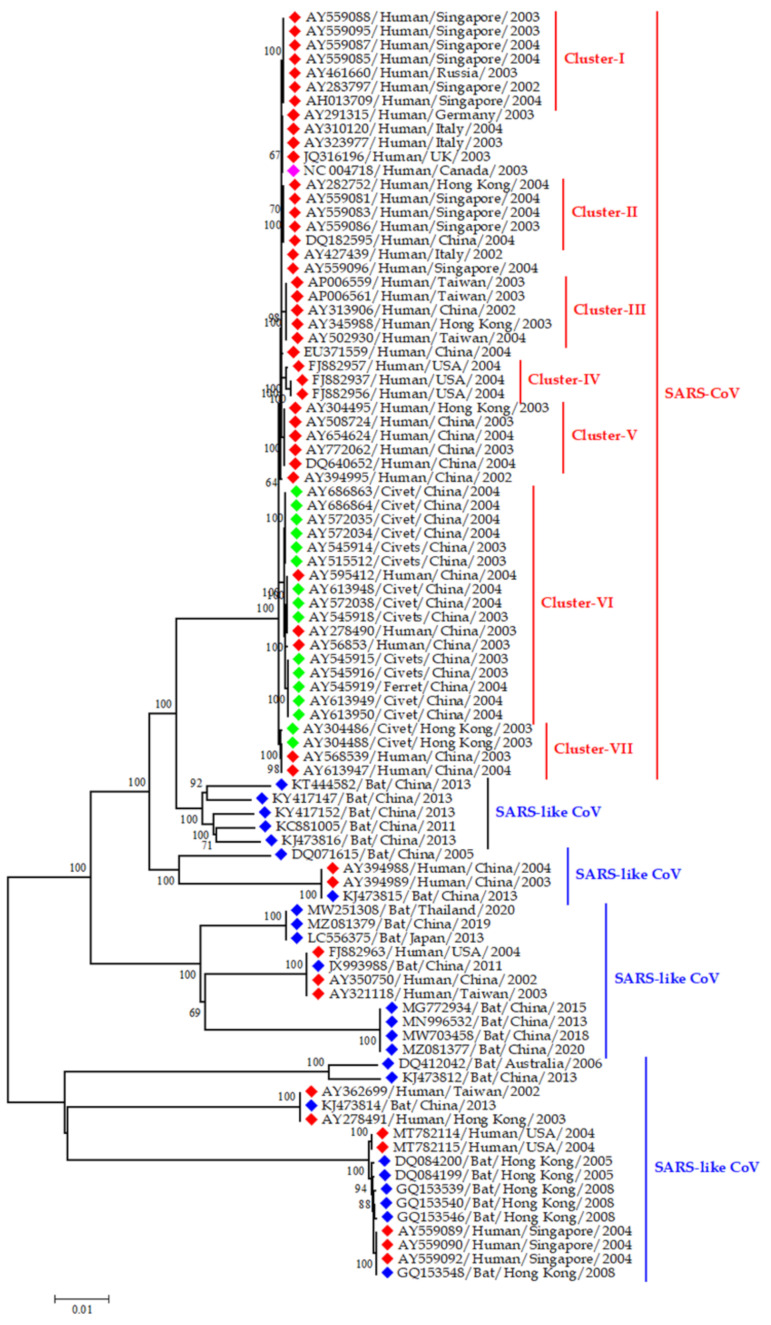
Phylogenetic analysis of SARS-CoVs from humans and SARS-like CoVs from animals; the red blocks indicate human strains; the blue blocks indicate bat strains, and the green blocks indicate sequences from civet and ferret. The pink block denotes the reference SARS sequence (NC004718) from the human. This phylogenetic tree representing the evolutionary relationship of representatives both SARS-CoV and SARS-CoV like viruses from human and animals like civet, and horse shoe bats. On the basis of time and space we selected the representative sequence of SARS-CoV and SARS-CoV like viruses in our phylogenetic analysis. The main purpose was to explain the evolutionally origin SARS-CoV and SARS-CoV like virus from human, civet and or bat.

Before the SARS outbreaks, the coronaviruses were not considered highly pathogenic for humans [34]. During the outbreak of SARS-CoV in 2002 and 2003, around 8000 cases were reported, with the death of 774 individuals from 37 countries around the world (Figure 6) [155]. Later, it was found that SARS-CoV was originated from Rhinolophus bats. Bat CoVs and SARS-CoV shared 99.8% nucleotide similarity. Then, the virus was transmitted to humans through intermediate hosts, masked palm civets [17] and raccoon dogs (Figure 7) [108].

After that, the MERS emerged in Saudi Arabia in September 2012 and produced 2562 cases with 881 deaths from 27 countries (Figure 6) (WHO, 2020). MERS-CoV was first detected in a man in Saudi Arabia in 2012. Later, it spread to Jordan and other countries outside the Arabian Peninsula [52,53,54]. The Vespertilionidae and Nycteridae families of bats were found to be the ancestor of MERS CoV [156]. In the case of MERS, the dromedary camel (mainly *C. dromedarius*) acted as an intermediate host [97]. However, it is challenging to know the exact route of the MERS virus from bats to camels; during the early 1980s, widespread cases in the Middle East and north and east Africa suggest that camel is a potential reservoir of MERS-CoV for at least 30 years [19]. Scientists found serological evidence, as well as viral RNA, of MERS-CoV in dromedary camels [50,55]. MERS cases are still found sporadically in the world.

**Figure 8:**
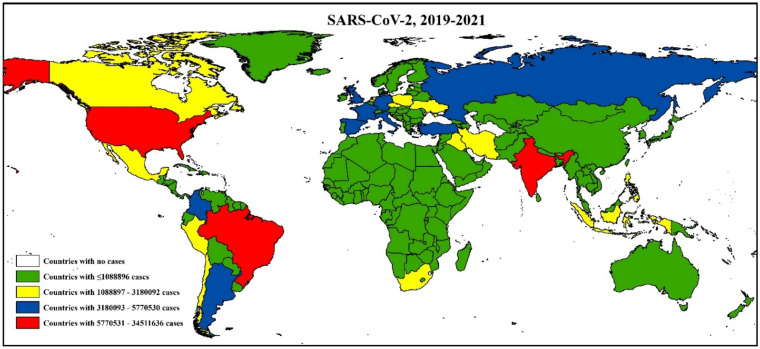
Distribution of SARS-CoV-2 cases around the world. The white areas have no cases, whereas the red marked regions have the highest number of cases.

The recent CoV, SARS-CoV-2, emerged in Wuhan, China, in December 2019 and rapidly spread worldwide. As of June 30, 2021, the virus has caused 183 million cases, with 39 million deaths after its emergence (Figure 8). Similar to SARS and MERS, SARS-CoV-2 was also originated from Rhinolophus bats [157]. The bat CoVs have almost 96.2% genetic resemblance with SARS-CoV-2 [158]. Initially, the pangolin was thought to have played a role in transmitting the virus to humans, but later, it was confirmed that pangolin itself contracts the virus from any other animal species along the trading pathway [98].

The nomenclature of SARS-CoV-2 was developed by a group of scientists and representatives from the WHO virus evolution working group, GISAID, Nexstrain, Pango, and virological and microbial nomenclature experts from different countries who recommended using the Greek alphabet Alpha 202012/01 GRY (B.1.1.7 + Q.x), Beta GH/501Y.V2 (B.1.351 + B.1.351.2 + B.1.351.3), Gamma GR/501Y.V3 (P.1 + P.1.x), Delta GK/478K.V1 (B.1.617.2 + AY.x), Eta G/484K.V3 (B.1.525), Iota GH/253G.V1 (B.1.526), Kappa G/452R.V3 (B.1.617.1), Lambda GR/452Q.V1 (C.37), and Mu GH (B.1.621 + B.1.621.1) based on the lineage pattern of the virus [159]. Moreover, along with traditional PCR-based virus detection methods, other methods, including radiologic imaging, ELISA, Lateral Flow Immunoassay (LFIA), neutralization assay, chemiluminescent assay, aptamer, CRISPR-Cas, molecularly imprinted polymer (MIP), Microarray, and Loop-mediated isothermal amplification (LAMP) based detection might be used to diagnose the existence of SARS-CoV-2 RNA [160].

## 4. Discussion

The CoVs had a long history from the year 1950 to 2019. In 1950, initially, the virus was identified among rodents, where cattle were the intermediate hosts. Later, the viruses were identified among bats and mice as natural hosts. The replication, mutation, and recombination rates of CoVs are moderate-to-high compared to those of other single-stranded RNA viruses. Both homologous and heterologous recombination events are expected for CoVs [18].

### 4.1. Emerging and Endemic CoVs Infection in Livestock and Companion Animals

A wide range of livestock CoVs produce different diseases in domesticated animals, but not all of them have zoonotic significance. The BCoV has a wide host range [39] and is genetically related to the human β-CoV OC43. HCoV-OC43 may originate from BCoV, or they might have a common ancestor [39]. Besides bovine CoVs, a critical pig pathogen, SADS-CoV might pose a similar risk to human health through transmitting from pigs or other intermediate hosts [161]. However, porcine CoVs have a very low (40.4–49.3%) nucleotide resemblance with the new pandemic pathogen, SARS-CoV-2. Thus, it is confirmed that pigs are not the source of SARS-CoV-2. However, there is a high similarity between virus binding residues between porcine angiotensin-converting enzyme 2 (ACE2) and human ACE2, so that SARS-CoV-2 can recognize porcine ACE2. This indicates the virus may have the capability to infect pigs, and that subsequent mutation with various porcine CoVs could produce a new strain with excess transmissibility. Though pigs are not susceptible to SARS-CoV-2 [23,157], cross-species transmission and mutations may occur with porcine CoVs. At present, vaccines are available for only TGEV and PEDV; however, their efficacy in the field is uncertain [162].

Additionally, canine CoV and porcine TGEV can exchange genes between them and cross the species barrier between cat and pig [74]. Moreover, CRCoV may have a common ancestor with bovine CoV, as they have nucleotide resemblance [76]. Few reports of recombinant canine CoVs are found. Camels are the reservoir of MERS-CoV but are less susceptible to SARS-CoV-2 [20]. Experimental studies regarding camels’ susceptibility to SARS-CoV-2 remain limited.

Several countries in the world documented human to the animal transmission of SARS-CoV-2 [163]. Furthermore, the emerging alpha variants, B.1.1.7 detection in pet animals, showed a gradual increase in the transmissibility and host range of the SARS-CoV-2 [157]. Therefore, extensive research on emergence, evolution, pathogenesis and immune responses is crucial for the effective control of future CoVs from animal sources. Currently, strict biosecurity and regular surveillance should be practiced in domestic animal farms.

### 4.2. Emerging CoVs in Wildlife

Bats are the ancestor of public health significant CoVs such as SARS, MERS, SARS-CoV-2, HCoV-229E [90,91,92], and CoVs significant for animal health i.e, SADS-CoV [46]. Similarly, pangolin was found to be infected by SARS-CoV-2 related CoVs [96]. Moreover, raccoon dogs and masked palm civets can act as intermediate hosts of SARS-like CoV strains [108]. The civet strains of SARS-like CoVs were closely related to human strains causing the SARS outbreak in 2002–2003 [164].

BCoV from cattle can be transmitted to other ruminants, different wildlife species, and vice versa [41,165,166,167]. Several captive wild animal species infections by SARS-CoV-2 occurred from the human. Even emerging variants as delta (B.1.617.2) have already been detected in wild animal species [157]. The wild animal species may help the evolution and transmission of CoV to other susceptible animal species. As a result, the virus can persist in nature and emerge from time to time. A considerable amount of deforestation, hunting, and farming causes wild animals to shift their habitats and food habits, consequently furthering viral adaption in new hosts and environments. Furthermore, common cold-causing organisms such as OC43, 229E, NL63, and HKU1 can infect non-human primates [138].

Similarly, marine mammals have their CoVs [141,144]. The marine γ-CoVs are highly similar to human coronaviruses, and they have similar morphology, genome organization, and replication [142,143]. With human effluent, there is an immense opportunity for SARS-CoV-2 to be released into the marine environment. SARS-CoV-2 nucleic acids have been detected in sewage and wastewater, which might occur through urban or agricultural runoff or in wastewater effluent [168,169]. This, in turn, can give rise to environmental contamination and subsequently can transmit to wild and aquatic animals. Therefore, wastewater effluent treatment is highly recommended to avoid viral spread [170]. Simultaneously, surveillance for SARS and other related CoVs and proactive viral discovery in wildlife is crucial to understand the origin and evolutionary history. For surveillance, RT-PCR based test can be used. However, as it will be expensive for resource-limited countries, there are options of using rapid antigen tests for screening a large number of samples [171]. Though some projects (e.g., PREDICT) were launched with this kind of surveillance in wildlife in some countries of the world [172], it needs to be spread to other hotspot regions. 

### 4.3. Emerging and Endemic CoVs Infection in Humans

The endemic human CoVs are OC43, 229E, NL63, and HKU1. These viruses are animal-borne, and the spillover events are already proven. Moreover, three other pandemic CoVs, SARS, MERS, and SARS-CoV-2, also originated from animals. SARS, MERS, and SARS-CoV-2 are considered emerging zoonotic viruses that have caused serious health consequences in humans. The SARS-CoV-2 was spread from a seafood and wild animal market in China [173,174]. Initially, most of the earliest cases have been infected from animal or environmental contacts, involved with the consumption of wild animals, and showing possible animal-to-human transmission. However, according to recent studies, human-to-human transmission of SARS-CoV-2 through respiratory droplets (coughing, sneezing, etc.) has been reported. In addition, hospital-acquired infection is a major hallmark of SARS, MERS, and SARS-CoV-2 [146].

The basic reproductive number of SARS-CoV-2 ranges from 1.173–3.5 [175], higher than that of SARS and MERS. Structural analysis of SARS-CoV-2 suggested that the virus uses the same cell receptor (ACE2) as SARS-CoV for entry, but due to higher affinity, SARS-CoV-2 could be more infectious to humans than SARS [176]. SARS-CoV-2 is changing through continuous mutations and gives rise to emerging variants of concern such as alpha (B.1.1.7), beta (B.1.351), and delta (B.1.617.2) [175]. After ripping through the European countries, the alpha-variant is now in decrease. The delta-variant is spreading rapidly and is responsible for the second and third waves in most nations [83]. SARS-CoV-2 cases and the number of deaths from different countries are continuously increasing day by day. Though all the countries have been affected by COVID-19, some specific regions in each country showed clustering of cases [177]. However, the current estimation depicting that mortality due to SARS-CoV-2 is comparatively higher than influenza. Dating back to the SARS-CoV epidemic in 2002–2003 and the MERS-CoV epidemic in 2012, they cause 10% and 35% mortality in humans, respectively [146,178].

Moreover, similar to influenza, the coronaviruses can cause rapid mutation and new strains through reassortment. Several variants of SARS-CoV-2 have already been detected in humans, animals, and the environment in various countries [28,118,179,180]. Thus, continuous genomic surveillance in human-animal–environmental interfaces is necessary to understand the molecular evolution and forecast upcoming pandemics by any CoVs. The scientific community is scared that the current pandemic might be turned into endemic coronavirus, producing occasional outbreaks globally. The research communities recommend making a universal vaccine effective against all types of coronaviruses [181] to encounter the future problem. Although researchers across the globe are desperately working to produce efficient vaccines against COVID-19, we already have seven vaccines currently using for vaccination, and around another 200 candidate vaccines are in a different stage of clinical trials [181]. The scientist uses their knowledge on the interaction between virus and immune system to produce a universal coronavirus vaccine [182]. Still, the expectation of a broad, adaptable candidate vaccine is highly ambitious, and the possibilities of getting these vaccines is questionable. Therefore, the existing practices of social distancing, wearing masks, and antiviral drugs therapy are needed to curb the infection.

## 5. Conclusions

Many mammalians and avian species, both domestic animals and wildlife, are susceptible to diverse CoV. Moreover, coronavirus strains cause epidemics and pandemics, significantly impacting public health, society, and the global economy. BCoVs have a common ancestor with buffalo CoV and CCoV. Similarly, porcine CoVs, especially SADS, has an affinity to species other than pigs. MERS-CoV is found in both camels and humans, though camels are affected less severely than humans. MERS-CoV cases are still occurring sporadically. In contrast, IBV is an important CoV for different avian species. Poultry companies are using a vaccine against IBV worldwide, but the emergence of novel serotypes makes it challenging to control. Moreover, FECV and CCoV recombine to produce FIPV. FIPV can infect other wild cats. BCoV-like viruses can also infect several wild ruminants. Among the seven HCoVs, four are endemic, having mild common cold-like infections, and three are emerging, zoonotic, and cause severe illness in humans. Rhinolopoid bats are the progenitors of SARS-CoV and SARS-CoV-2 related coronaviruses. To prevent the recurrent outbreak of novel CoVs worldwide, we recommend developing a universal vaccine against CoV to protect humans. Furthermore, we recommend strengthening animal surveillance and early warning system to prevent future CoV outbreaks from animal sources.

## Figures and Tables

**Table 1:** Keywords for searching published literature in different databases.

Term	Keywords
Descriptive term	Prevalence OR Incidence OR Frequency OR Occurrence OR Infection OR Detection OR Identification OR Isolation OR Characterization OR Investigation OR Survey OR Rate
Outcome term	Coronavirus OR α-CoV OR β-CoV OR γ-CoV OR δ-CoV OR MERS OR SARS-CoV OR COVID-19 OR SARS-CoV-2 OR HKU2 OR CCOV OR CRCoV OR FECV OR FIPV OR BCoV OR HCOC43 OR HC229E OR HCNL63 OR HKU1 OR TGEV OR PEDV OR PRCV OR SADS-CoV OR PHEV OR PDCoV OR IBV
Population term	Human OR Animal OR Mammals OR Birds OR Avian OR Poultry OR Turkey OR Chicken OR Goose OR Pheasant OR Domestic animals OR Bovine OR Cattle OR Calf OR Equine OR Horse OR Pig OR Camel OR Canine OR Feline OR Dog OR Cat OR Wild animals OR Tiger OR Lion OR Mink OR Rodents OR Bat OR Pangolin OR Monkey OR Mice OR Rat OR Ferret OR Guinea pig OR Masked Civet

**Table 2:** Coronavirus diversity in domestic animals and birds.

Host	Disease	Virus Type	System Affected
Cattle	Bovine CoV	βCoV	Respiratory, Digestive
Buffalo	Bubaline CoVs	βCoV	Respiratory, Digestive
Pig	TGEV	αCoV	Respiratory, Digestive
Pig	PRCV	αCoV	Respiratory
Pig	SADS	αCoV	Digestive
Pig	PEDV	αCoV	Digestive
Pig	PHEV	βCoV	Respiratory, Digestive, Nervous
Pig	PDCoV	δCoV	Digestive
Camel	MERS	βCoV	Respiratory
Camel	β1-HKU23-CoVs	βCoV	Respiratory
Camel	Camelid α-CoV	αCoV	Respiratory
Alpaca	Alpaca CoV	αCoV	Respiratory
Horse	Equine CoV	βCoV	Digestive
Turkey	TCoV	ϒCoV	Digestive
Chicken	IBV	ϒCoV	Respiratory, Urinary, Reproductive
Bulbul	BuCoV HKU11	δ-CoV	
Thrush	ThCoV HKU12	δ-CoV	
Munia	MunCoV HKU13	δ-CoV	
White-eye	HKU16	δ-CoV	
Sparrow	HKU17	δ-CoV	
Magpie robin	HKU18	δ-CoV	
Night heron	HKU19	δ-CoV	
Wigeon	HKU20	δ-CoV	
Common moorhen	HKU21	δ-CoV	
Quail	Quail CoV	δ-CoV	
Dog	CCoV	αCoV	Respiratory
Dog	CRCoV	βCoV	Respiratory
Dog	SARS CoV-2	βCoV	Respiratory
Cat	SARS CoV-2	βCoV	Respiratory
Cat	FIPV	αCoV	Monocyte
Cat	FECV	αCoV	Digestive
